# Placental ion channels: potential target of chemical exposure

**DOI:** 10.1093/biolre/ioac186

**Published:** 2022-09-29

**Authors:** Yi Zhao, Markku Pasanen, Jaana Rysä

**Affiliations:** Department of Obstetrics, The First Affiliated Hospital of China Medical University, Shenyang, Liaoning, China; School of Pharmacy, University of Eastern Finland, Kuopio, Finland; School of Pharmacy, University of Eastern Finland, Kuopio, Finland

**Keywords:** placenta, ion channel, trophoblast, smoking, chemical exposure, pharmaceuticals

## Abstract

The placenta is an important organ for the exchange of substances between the fetus and the mother, hormone secretion, and fetoplacental immunological defense. Placenta has an organ-specific distribution of ion channels and trophoblasts, and placental vessels express a large number of ion channels. Several placental housekeeping activities and pregnancy complications are at least partly controlled by ion channels, which are playing an important role in regulating hormone secretion, trophoblastic homeostasis, ion transport, and vasomotor activity. The function of several placental ion channels (Na, Ca, and Cl ion channels, cation channel, nicotinic acetylcholine receptors, and aquaporin-1) is known to be influenced by chemical exposure, i.e., their responses to different chemicals have been tested and confirmed in experimental models. Here, we review the possibility that placental ion channels are targets of toxicological concern in terms of placental function, fetal growth, and development.

## Teaser

Ion channels participate in regulating key placental functions. The effects of chemicals affecting ion channels have rarely been studied although theoretically these compounds could cause pregnancy complications.

## Introduction

The human placenta is a complex organ between the mother and the fetus. The primary function of the placenta is the exchange of nutrients, gases, and other substances between the mother and the fetus. Placenta also serves as a fetal nutrient storage site and produces hormones such as estrogen and progesterone, placental lactogen, and human chorionic gonadotropin (hCG). In addition, the placenta has defensive and immunomodulatory capabilities. Placenta has the semipermeability of membrane barrier selection, and it synthesizes immunomodulatory factors such as cytokines to protect the fetus. Finally, the placenta also regulates fetal metabolism, especially during the early and mid-term stages of pregnancy [1, 2].

Exposure to chemicals during pregnancy, such as maternal smoking, medication, consumer chemical products, and through environmental pollution, may affect the placental function and, in the worst case, may even result in an adverse pregnancy outcome [3]. Many chemical compounds can cross the placental barrier and gain further access to the fetal compartment [4]. The transfer is conducted through the chorionic membrane barrier, a polarized epithelial structure, which develops from the syncytiotrophoblast cells and has several specific transport mechanisms including ion channels and transporters [5]. Ion channels are specialized membrane-spanning, pore-forming protein macromolecules, which facilitate the rapid transmembrane transfer of water-soluble molecules such as inorganic ions [6]. Owing to the different functions in different tissues and organs, the distribution of ion channels and subtypes tend to be organ specific. Although the activation of an ion channel is selective for its particular ions, its physiological consequences can be diverse.

Placental housekeeping activities are susceptible to chemicals and at least partly controlled by ion channels [5]. Consequently, these channels have great physiological and pathological significance. The objective of this review was to provide an update and comprehensive insight into placental ion channels. We review the information on expression of ion channels in human placental tissue and primary cells. Furthermore, we highlight the possibility that placental ion channels are targets of toxicological concern in terms of placental function, fetal growth, and development based on studies obtained in different placental platforms.

## Placental ion channels

Ion channels have two major characteristics: the first is selectivity––one type of channel preferentially passes certain ions, whereas other ions cannot easily pass through that channel. The second characteristic is an ability to switch; ion channels exist in two states, open and closed states. Ion channels can be divided into various ion channel subtypes depending on the type of ion and activation [6]. Human placenta expresses a large number of ion channels. The data on ion channels in human placental tissue and primary placental cells are shown in Table 1. The studies include placental tissue or primary cells from all trimesters of pregnancy, and also from abnormal pregnancies. For many ion channel types, the data on the human placenta are limited to mRNA expression studies. Among the most studied ion channels in placental platforms are nicotine acetylcholine receptors (nAchR), transient receptor potential channels (TRPs) TRPP2, TRPV5, and TRPV6, aquaporins (AQP) 3, 8, and 9, and connexin 43 (Cx43).

**Table 1:** The distribution of ion channels in human placental tissue and primary human placental cells

			Expression in placenta			
	Name	Gene name	mRNA^*^	Protein^*^^*^	Localization^*^^*^^*^	References
Ligand-gated ion channels						
Epithelial sodium channels						
	ENaCα	*SCNN1A*	+	+	Cytotrophoblasts, syncytiotrophoblasts	[7–11]
	ENaCβ	*SCNN1B*	+	+	Syncytiotrophoblasts	[7,10]
	ENaCγ	*SCNN1C*	+	+	Syncytiotrophoblasts	[7,9,10]
GABA-type receptors						
	GABA A	*GABRP*	+	+	Cytotrophoblasts, syncytiotrophoblasts.	[12–14]
Inositol 1,4,5-trisphosphate receptors (IP3R)						
	IP3R1	*ITPR1*	+	−	−	[15]
	IP3R2	*ITPR2*	+	−	−	[15]
	IP3R3	*ITPR3*	+	−	−	[15]
Nicotine acetylcholine receptors						
	nAchRα1 subunit	*CHRNA1*	+	−	−	[16]
	nAchRα2 subunit	*CHRNA2*	+	+	Syncytiotrophoblasts, villi	[16–19]
	nAchRα3 subunit	*CHRNA3*	+	+	Syncytiotrophoblasts, blood vessels	[16–19]
	nAchRα4 subunit	*CHRNA4*	+	+	Syncytiotrophoblasts, blood vessels	[16–19]
	nAchRα5 subunit	*CHRNA5*	+	+	Syncytiotrophoblasts, blood vessels	[16–19]
	nAchRα6 subunit	*CHRNA6*	+	+	−	[16,17,19]
	nAchRα7 subunit	*CHRNA7*	+	+	Cytotrophoblasts, syncytiotrophoblasts, villi, blood vessels	[17–21]
	nAchRα9 subunit	*CHRNA9*	+	+	Syncytiotrophoblasts, villi, blood vessels	[16–19]
	nAchRα10 subunit	*CHRNA10*	+	+	Syncytiotrophoblasts, blood vessels	[16,17,19]
	nAchRβ1 subunit	*CHRNB1*	+	+	Villi	[16–18,22]
	nAchRβ2 subunit	*CHRNB2*	+	+	Villi	[16–18]
	nAchRβ3 subunit	*CHRNB3*	+	−	−	[16]
	nAchRβ4 subunit	*CHRNB4*	+	−	−	[16,17]
	nAchRδ subunit	*CHRND*	+	+	Villi	[16–18]
	nAchRγ subunit	*CHRNG*	+	−	−	[16]
	nAchRε subunit	*CHRNE*	+	−	−	[16,17]
P2X receptors						
	P2X1	*P2RX1*	+	−	Blood vessels	[23]
	P2X4	*P2RX4*	+	+	Cytotrophoblasts, blood vessels	[23–25]
	P2X5	*P2RX5*	+	−	Blood vessels	[23]
	P2X6	*P2RX6*	+	−	Blood vessels	[23]
	P2R7	*P2RX7*	+	+	Cytotrophoblasts, blood vessel	[23,24]
ZAC						
	ZAC	*ZACN*	+	−		[26]
Potassium channels						
Calcium- and sodium-activated potassium channels						
	K_Ca_1.1	KCNMA1 KCaα	+	+	Blood vessels	[27–29]
		KCNMB1 BKCaβ	+	+	Blood vessels	[30]
	K_Ca_2.1	KCNN1 SKCa1, SK1	+	+	Blood vessels	[31]
	K_Ca_2.3	KCNN3 SKCa3,	+	−	Blood vessels	[28]
	K_Ca_3.1	KCNN4 IKCa, IK1, IKCa3.1, SKCa4	+	+	Cytotrophoblasts, blood vessels	[28,31,32]
Inwardly rectifying potassium channels						
	K_ir_2.1	*KCNJ2*	+	+	Syncytiotrophoblasts, blood vessels	[33–35]
	K_ir_6.1	*KCNJ8*	+	+	Syncytiotrophoblasts, blood vessels	[29,34,36]
	K_ir_6.2	*KCNJ11*	+	−	Blood vessels	[36]
Two P domain potassium channels						
	K_2p_2.1 (TWIK)	*KCNK1*	+	−	−	[37]
	K_2p_2.1 (TREK1)	*KCNK2*	+	+	Cytotrophoblasts, syncytiotrophoblasts	[33,38]
	K_2p_3.1 (TASK1)	*KCNK1*	+	+	Cytotrophoblasts, syncytiotrophoblasts, blood vessels	[29,33,38]
	K_2p_4.1 (TRAAK)	*KCNK4*	+	−	−	(Lesage, Maingret and Lazdunski, 2000)
	K_2p_5.1 (TASK2)	*TASK2* *KCNK5*	+	−	−	[38]
	K_2p_6.1 (TWIK2)	*KCNK6*	−	+	Blood vessels	[39]
	K_2p_7.1 (TWIK3)	*KCNK7*	−	+	Blood vessels	[39]
	K_2p_9.1 (TASK3)	*KCNK9*	−	+	Blood vessels	[39]
	K_2p_13.1 (TASK5)	*KCNK15*	+	−	−	[38]
	K_2p_17.1 (TALK2, TASK4)	*KCNK17*	+	−	−	[38,40]
	K_2p_18.1 (TRESK-2)	*KCNK18*	+	−	−	[41]
Voltage-gated potassium channels						
	Kv1.5	*KCNA5*	+	+	Blood vessels	[27]
	Kv2.1	*KCNB1*	−	+	Syncytiotrophoblasts, blood vessels	[27,29,33,34]
	Kv3.1	*KCNC1*	−	+	Blood vessels	[27]
		*KCNE1*	+	+	−	[42–44]
		*KCNE2*	+			[43,44]
		*KCNE3*	+	−	−	[42–44]
		*KCNE4*	+	−	−	[42,44]
		*KCNE5*	+	−	Syncytiotrophoblasts, blood vessels	[42–44]
	Kv6.2	*KCNE6*	−	+		[45]
	Kv7.1	KCNQ1	+	+	Blood vessels	[42–44,46,47]
	Kv7.2	KCNQ2	+	+	Blood vessels	[43,44,47]
	Kv7.3	KCNQ3	+	+	Syncytiotrophoblasts, blood vessels	[42–44,46,47]
	Kv7.4	KCNQ4	+	+	Blood vessels	[43,44,46,47]
	Kv7.5	KCNQ5	+	+	Blood vessels	[42,43,46,47]
	Kv9.3	KCNS3	+	+	Syncytiotrophoblasts, blood vessels	[29,34,48]
Ryanodine receptors						
	RyR1	*RYR1*	+	−	Cytotrophoblasts, syncytiotrophoblasts	[15,49]
	RyR2	*RYR2*	+	−	−	[15]
	RyR3	*RYR3*	+	−	Cytotrophoblasts, syncytiotrophoblasts	[15,49]
Transient receptor potential cation channels						
	TRPC1	*TRPC1*	+	+	−	[50]
	TRPC3	*TRPC3*	+	−	Cytotrophoblasts, syncytiotrophoblasts	[50]
	TRPC4	*TRPC4*	+	+	Cytotrophoblasts, syncytiotrophoblasts	[50]
	TRPC5	*TRPC5*	+	−	−	[50]
	TRPC6	*TRPC6*	+	+	Syncytiotrophoblasts	[50]
	TRPM2	*TRPM2*	+			[51]
	TRPM4	*TRPM4*	+			[51]
	TRPM7	*TRPM7*	+			[51]
TRPP (polycystin) family						
	TRPP1	*PKD2*	+	+	Trophoblasts	[52]
	TRPP2	*PKD2L1*	+	+	Syncytiotrophoblasts, trophoblasts	[52–59]
TRPV (Vanilloid) family						
	TRPV1	*TRPV1*	+	+	Cytotrophoblasts, syncytiotrophoblasts	[60,61]
	TRPV4	*TRPV4*	+	+	Cytotrophoblasts, syncytiotrophoblasts	[62]
	TRPV5	*TRPV5 (*ECaC1*, *CaT2*)*	+	+	Syncytiotrophoblasts, villi	[15,63–65]
	TRPV6	*TRPV6 (*ECaC2, CaT1*)*	+	+	Syncytiotrophoblasts, villi	[15,63,65,66]
Voltage-gated calcium channels						
	Ca_V_1.1	*CACNA1S*	+	+	Syncytiotrophoblasts, villi	[65]
	Ca_V_1.2	*CACNA1C*	+	+	Syncytiotrophoblasts, villi, blood vessels	[39,63,65]
	Ca_V_1.3	*CACNA1D*	+	−	−	[63]
Voltage-gated sodium channels						
	Na_v_1.8	* SCN10A*	+	−	−	[67]
Other ion channels						
Aquaporins						
	AQP1	*AQP1*	+	+	Cytotrophoblasts, villi blood vessels	[68–71]
	AQP2	*AQP2*	−	+	Syncytiotrophoblasts, trophoblasts	[72]
	AQP3	*AQP3*	+	+	Cytotrophoblasts, syncytiotrophobasts, villi	[68–71,73–77]
	AQP4	*AQP4*	+	+	Syncytiotrophoblasts, villi, endothelial cells	[68,71,78,79]
	AQP5	*AQP5*	+	−	Villi	[68]
	AQP8	*AQP8*	+	+	Cytotrophoblasts, syncytiotrophoblasts, villi	[68,70,71,80–83]
	AQP9	*AQP9*	+	+	Cytotrophoblasts, syncytiotrophoblasts, villi	[68,70,71,73,80,81,83–88]
	AQP11		+	−	Villi	[68]
Chloride channels						
ClC (CLC)-family						
	CIC-1	*CLCN1*	+	+	Trophoblasts	[89]
	CIC-3	*CLCN2*	+	+	Cytotrophoblasts, syncytiotrophoblasts, trophoblasts	[90,91]
	CIC-4	*CLCN4*	+	+	Trophoblasts, villi	[89]
	CIC-5	*CLCN5*	+	+	Trophoblasts, villi	[89,91]
CFTR-family						
	CFTR	*CFTR*	+	+	−	[11,92–95]
Maxi chloride channels						
	Maxi-Cl^−^	#	−	+	Syncytiotrophoblasts, trophoblasts	[96]
Connexins and pannexins						
	Cx26	*GJB2*	+		Syncytiotrophoblasts	[97]
	Cx31	*GJB3*	+		Syncytiotrophoblasts	[97]
	Cx32	*GJB1*	+	+	Cytotrophoblasts, syncytiotrophoblasts, trophoblasts	[97–99]
	Cx37	*GJA4*	+		Syncytiotrophoblasts	[97,99]
	Cx40	*GJA5*	+	+	Cytotrophoblasts, syncytiotrophoblasts, endothelial cells	[97,99–102]
	Cx43	*GJA1*	+	+	Cytotrophoblasts, syncytiotrophoblasts, trophoblasts, villi	[97–100,102–111]
	Cx45	*GJC1*	+	+	Syncytiotrophoblasts	[97,99]
	Cx46	*GJA3*	+		Syncytiotrophoblasts	[97]
	Px1	*Panx1*	+	+	Syncytiotrophoblasts	[97,112]
	Px2	*Panx2*	+		Syncytiotrophoblasts	[97]
	Px3	*Panx3*	+		Syncytiotrophoblasts, trophoblasts, villi	[97,105]
Piezo channels						
	Piezo1	*PIEZO1*	+		Endothelial cells	[113]
Orai channels						
	Orai1	*Orai1*	+		−	[114]

^*^In situ hybridization, Northern Blot, RT-PCR were used to measure mRNA levels, ^*^^*^western blot to detect protein and ^*^^*^^*^immunohistochemistry, immunocytochemistry and immunofluorescence as well as cell fractions and vesicles were used to report localization of ion channels. ^#^A complex with solute carrier organic anion transporter family member 2A1 as a core pore-forming component and two auxiliary regulatory proteins, annexin A2 and S100 calcium binding protein A10 [115].

CaV, voltage-gated calcium channel; CFTR, cystic fibrosis transmembrane conductance regulator (ATP-binding cassette sub-family C, member 7); CLC, chloride channel; Cx, connexin; K_2P_, two-pore domain potassium channel; KCa, calcium-activated potassium channel; K_ir_, inwardly rectifying potassium channel; nAchR, nicotinic acetylcholine receptor; Na_V_, voltage-gated sodium channel; P2X, purinergic receptor P2X Px, pannexin; Ryr, ryanodine receptor; TRPM, transient receptor potential cation channel subfamily M; TRPP, transient receptor potential cation channel subfamily P; TRPV, transient receptor potential cation channel subfamily V; ZAC, zinc-activated channel.

### Calcium channels

Calcium transfer in the human placenta mainly occurs through the syncytiotrophoblasts and calcium channels are the most widely distributed type of ion channel in the placenta [116,117]. In placenta, voltage-gated L-type Ca channel isoforms (Ca_v_1.1, Ca_v_1.2, and Ca_v_1.3), and Cav1.4 have been detected in syncytiotrophoblasts [63,64], where these channels would also have a role in cell signaling and protein secretion [117]. In placental blood vessels, voltage-gated L-type Ca channels are mainly involved in regulating the hypoxic fetoplacental vasoconstriction [118] and in syncytiotrophoblasts, these channels would have a role in also in cell signaling and hormone secretion in addition to cellular Ca^2+^ entry [63–65].

### Transient receptor potential channels

Human placenta expresses several isoforms of TRPs [116], of which transient receptor potential cation channel subfamily C (TRPCs) may have a role in store-operated calcium entry (i.e., activated by intracellular calcium released particularly from the endoplasmic reticulum store) in the placenta. In addition, TRPV5 and − 6 are calcium selective channels that seem to be strongly involved in the calcium transport from the mother to the fetus [15]. In fact, mutations in TRPV6 coding gene were shown to interfere with calcium transport through the placenta and cause fetal calcium deficiency, hyperparathyroidism, and metabolic bone disease [119]. In addition, a non-selective, voltage-dependent TRPP2 channel (polycystin-2) is located at the apical membrane of the syncytiotrophoblasts with a high permeability to Ca^2+^ but also permeable to Na^+^ and K^+^ [53]. It may be important for Ca^2+^ transport but also for regulation of other ions transport in the placenta [53–56].

### Chloride channels

Placental chloride channels are important for chloride transport at the plasma membrane. The members of chloride intracellular channel (CIC) proteins are ubiquitously expressed and involved in the transplacental passage of chloride but also in the regulation of several other cellular processes including proliferation, differentiation, and apoptosis although their role is not completely understood [89–91]. However, the expression of these chloride channels is increased in placentae of pre-eclamptic (PE) and intra-uterine growth retardation pregnancies (IUGR), but it remains to be studied whether they contribute to pathological processes of these conditions [65,90]. Maxi-chloride channel is a complex with solute carrier organic anion transporter family member 2A1 (SLCO2A1) as pore-forming component and two auxiliary regulatory proteins, annexin 2 and S100 calcium binding protein A10 (S100A10) [115] Maxi-chloride channel has been shown to be regulated by arachidonic acid, fatty acids and steroid hormones [120]. In addition to chloride, the maxi-chloride channel is permeable to amino acids and it may have a role in placental volume regulation [121].

### Potassium channels

Potassium channels play important physiological roles in the human placenta including membrane permeability to K^+^ ions, the control of fetoplacental blood flow, and hormone secretion [122]. With respect to the identified potassium channels, many members of voltage-gated, calcium and sodium -activated and 2-pore domain potassium channels participate in hormone secretion in the placenta [33,36,42,46,48,123–125]. Many K-channels distributed in the placental blood vessels are oxygen-sensitive and participate in controlling the vascular tone of the placental blood vessels [42,125,126]. In fetal growth restriction, gene expression of voltage dependent potassium channel K_V_9.3 and K_V_2.1 was increased in placental tissue and veins, respectively [34]. Similarly, the expression of Kv2.1, inwardly rectifying potassium channel Kir2.1 increased in the basal membrane of placentas from PE and IUGR pregnancies compared to placentas of healthy pregnancies, where these channels were mainly present in the apical membrane [33]. In addition, during the differentiation of cultured human trophoblasts, the expression of K_V_7 channel subunits (KCNQ1, KCNE1, KCNE3, and KCNE5) was decreased by hypoxia and induced in an oxygen-rich environment [42]. Altogether, the changes in the expression of potassium channels in vasculature and trophoblasts, and their localization between apical and basal membranes in pathological conditions such as PE and IUGR [33,34,42] suggest that these ion channels may have a role in regulating placental physiology.

### Other ion channels

Several subunits and receptor subtypes of nicotinic acetylcholine receptors nAChR are expressed in the placenta. In PE, expression of several mAChR subunits is dysregulated [17,18]. Placenta also expresses an epithelial sodium channel (ENaC), which is located in the apical membrane of cytotrophoblasts [7]. The expression of ENaC is regulated by aldosterone and a reduced amount of ENaC is reported in PE [127]. In addition to control of the intracellular flow of sodium ions, ENaC may promote cell migration in the placenta [127,128]. In addition, the expression of π subunit of the ion channel gamma-aminobutyric acid (GABA) A receptor (GABRP) is also increased in the placentas of patients with PE. In HTR-8/SVneo trophoblastic cells, GABRP was shown to promote apoptosis and inhibit the invasion of trophoblastic cells that could have a role in the onset of PE [12]. Furthermore, a special type of ATP-activated ion channels called the purinergic receptors are expressed in human cytotrophoblast cells. Ligand-gated P2X4, P2X7, and G protein-coupled P2Y2 and P2Y6 have been reported to modulate the intracellular concentration of Ca^2+^ and K^+^ efflux in cytotrophoblasts [24,25] although their significance in the control of placental electrolyte transport is not studied in detail and consequently their role in the placenta is not yet known. In addition, several aquaporins (AQPs), water channels that also have an additional ion channel function, are expressed in the human placenta on placental trophoblasts, chorionic villi, and fetal membrane [129]. In the placenta, as water channels, they have a role as a regulator of maternal-fetal fluid flow but the ion channel roles remain to be defined [130].

## The effect of chemical exposure on placental ion channels

### Chemicals in tobacco products

Effects of chemical exposure on ion channels have been studied in multiple placental platforms. We have reviewed the findings on experimental models including cell lines and experimental animal studies in Table 2. Smoking during pregnancy is the most common type of chemical exposure to placenta, e.g., leading to disturbed trophoblast morphology and invasion, reduction in placental development, and ultimately retarded fetal growth [131,132]. Studies with the term placentas have indicated that cigarette smoke and nicotine can dysregulate levels of nAchR subtypes in the placenta [16,18,133–137]. In addition, nicotine competes with endogenous acetylcholine for binding to nAChRs [138]. In fact, nAChRs in rat trophoblast cells are responsive at nicotine concentrations similar to nicotine plasma levels detected among moderate to heavy cigarette smokers [16,19,137]. One of the possible mechanisms by which nicotine impairs placental function could be increased endoplasmic reticulum stress via nAChR [138]. In addition, nicotine has been shown to suppress placental cytokine production mediated through the nAChR pathway [136].

**Table 2:** Effects of smoking and chemical exposure on ion channels obtained in different placental platforms

Chemical	Ion channel	Material	Experimental results	References
Cigarette smoke	nAchR	Full term human placenta	Increased expression of nAChRα9 and β1 subunits	[18]
Nicotine	nAchR	Full term human placenta	Increased levels of nAChRα9 subunit and decreased levels of nAChRδ	[16]
Nicotine	nAchR	HTR-8/SVneo cells	Suppressed invasiveness of human trophoblasts by downregulation of CXCL12 expression through nAChRα7 subunit	[134]
Nicotine	nAchR	BeWo cells	Increased expression of nAChRα9 subunit	[135]
Nicotine	nAchR	Rat placental explants	Regulated increased expression of nAChRα7 subunit in placenta after lipopolysaccharide treatment	[133]
Nicotine	nAchR	In vivo in rats, rat trophoblast Rcho-1 cell line	Increased expression of nAChRα4 subunit	[137]
Nicotine	nAchR	Rat trophoblast Rcho-1 cell line	Increased endoplasmic reticulum stress via nAChR.	[138]
Nicotine	nAchR	Primary human placental cells	Inhibited cytokine production via nAChR pathway	[136]
Aflatoxin B1	TRPs	Placental JEG-3 cells	Increased mRNA levels of TRP subtypes C3, C4, C6, V5, P2 TRPC3 mediated AFB1 induced increase in COX-2 expression	[139]
Zeranol	TRPs	Placental JEG-3 cells	Increased mRNA levels of TRP subtypes C3, C6, P2 TRPC3 mediated zeranol induced increase in COX-2 expression	[140]
Bisphenol A, octylphenol	TRPV6	Mouse placenta	Decreased mRNA levels of TRPV6	[141]
Bisphenol A	ENaCα	Mouse fetal membrane	Decreased ENaCα protein levels	[142]
Aroclor 1254	AQP1	Mouse placenta Human HTR8 cells	Reduced AQP1 protein levels	[143]
ROS	TRPP2	Human syncytiotrophoblasts	Inhibited TRPP2 activity	[58]
Aldosterone	ENaC	BeWo cells	Modulated ENaC currents	[128,144]
Aldosterone	ENaCα	Human HTR8/SVneo cell line	Upregulated ENaCα protein expression ENaC activity was important for trophoblast cells invasion	[8]
Prolactin, hGC	ENaCα	Human HTR8/SVneo cell line	Upregulated ENaCα protein expression	[8]
Verapamil, nifedipine	Calcium channel	Human first trimester placental tissue	Inhibited GnRH-stimulated hCG secretion	[145]
17-beta-estradiol, tamoxifen	Maxi-Cl- channel	Human placental apical membrane vesicles	Steroid hormones may regulate transplacental chloride transport	[146]
Bicuculline	GABA type A receptor	Human first trimester trophoblasts	Inhibited hCG secretion	[13]
Capsasin	TRPV1	Human cytotrophoblasts	Inhibited hCG secretion Impaired the spontaneous in vitro differentiation of cytotrophoblasts into syncytiotrophoblasts	[60]
Leptin	AQP9	Human placental explants	Upregulated AQP9 expression	[84]

CXCL12, C-X-C Motif Chemokine Ligand 12; GnRH, Gonadotropin-releasing hormone; JEG-3, Human choriocarcinoma cell line; nAchR, Nicotinic acetylcholine receptor; TRPV5/6, Transient receptor potential cation channel subfamily V member 5/6.

In addition, heavy metals such as cadmium, which are also present in tobacco smoke, can inhibit placental leptin synthesis, partly explaining the endocrine-disrupting effects of cadmium [147]. In a study using a human embryonic kidney HEK293 cell line, cadmium significantly inhibited calcium ion flow into the cells, and this was attributed to a competitive inhibition of two important ion channels TRPV5 and TRPV6 [148], both of which are important in fetal placental calcium transport as well [149], indicating that cadmium could directly affect fetal bone development via these ion channels.

### Environmental contaminants

Several common environmental pollutants such as dichlorodiphenylethylene (DDE), bisphenol A, brominated flame retardants, polychlorinated biphenyls, and fungal metabolites such as aflatoxins can be detected in the placenta [3]. Studies with the JEG-3 cell line demonstrated that endocrine-disrupting chemicals (EDCs), aflatoxin B1, and zeranol can significantly increase the mRNA levels of TRPC3 ion channel and increase intracellular calcium levels [139,140]. In addition, the experiments in pregnant mice demonstrated that bisphenol A and octylphenol significantly reduced placental TRPV6 mRNA levels, and furthermore disturbed fetal bone development in mice [141]. In another study, after intervention with bisphenol A, the expression of epithelial sodium channel alpha (ENaCα) protein in the decidua was significantly down-regulated detected by immunohistochemistry [142]. Thus, it has been suggested that exposure to BPA leads to impaired decidualization through a reduced serum glucocorticoid-induced kinase 1-mediated downregulation of ENACα [142,150]. In epidemiological studies, polychlorinated biphenyls (PCB) and other chlorides have been linked with serious adverse effects on maternal health and fetal development including growth restriction [151] impaired immune response [152] and neurobehavioral deficits [153] in the child. Finally, there is convincing evidence that aquaporin 1 (AQP1) is involved in the production of fetal amniotic fluid [154]. In the placenta of pregnant mice exposed to PCB, the protein expression of placental AQP1 was significantly downregulated in comparison to normal wild-type mice, and the amount of amniotic fluid was significantly increased [143].

### Pharmaceuticals

Several well-known pharmaceuticals even act via ion channels and some of them have been shown to affect placental function. A class of antihypertensive drugs, i.e., dihydropyridines have been shown to stimulate the secretion of hCG via calcium channels. Inhibited hCG secretion was also seen in response to capsaisin in human primary cytotrophoblasts, where capsaisin impaired differentiation of cytotrophoblasts into syncytiotrophoblasts by activating TRPV1 [60]. In addition, also GABA-A receptor agonis bicuculline can inhibit hCG secretion [13]. On the other hand, hCG, prolactin, and aldosterone were able to upregulate ENaCα protein expression in human extravillous trophoblast cell line [8].

Placenta is a highly active endocrine organ, and several ion channels are regulated by hormones. For example, 17β-estradiol (and tamoxifen) have been shown to regulate the Maxi-chloride channel in apical membranes from human placental syncytiotrophoblasts [146]. Furthermore, exogenous progesterone was shown to upregulate TRPV5 and TRPV6 mRNAs in ovine placentome [155] and an energy metabolism-regulating hormone leptin upregulated AQP9-expression in human trophoblast explants [84]. Finally, human term cytotrophoblast expresses the mineralocorticoid-responsive genes including ENaCα ja ENaCγ subunits [9] and it has been shown that aldosterone can promote cell migration via ENaC in BeWo cells [128,144].

## Concluding remarks

A large number of ion channels are distributed in the placenta where they have several important functions (1) to regulate the synthesis and secretion of hormones, (2) to ensure homeostasis of trophoblasts, (3) to control the transport of trace elements between mother and fetus, and (4) to regulate vascular contraction and relaxation. It has been confirmed that the placental ion channels exposed to chemicals can undergo functional and quantitative changes, which in turn could affect the normal function of the placenta and the growth and development of the fetus. However, so far, there are rather few reports on the effects of the chemical compounds on placental ion channels. The responses of placental ion channels to chemical exposure, which may be either direct or indirect, can potentially lead to pregnancy complications such as abortion, premature delivery, fetal growth restriction, fetal development abnormalities*, etc.* Further focused investigations using placental platforms are needed to clarify the potential role of chemical-induced ion channels medicated effects on placental housekeeping functions and whether pharmaceuticals acting *via* ion channels can become potential therapies in selected obstetric complications.

## Conflicts of interest

The authors have declared that no conflict of interest exists.

## Authors’ contributions

All authors contributed to the study, and read and approved the submitted version.
